# Advice to Young Doctors; Sick-Room Manners

**Published:** 1904-12

**Authors:** 


### ADVICE TO YOUNG DOCTORS; SICK-ROOM MANNERS.

We once heard a lady, the daughter of a prominent physician, and with exceptional advantages in other ways for forming an opinion, comment upon the manners of physicians in general in the sick-room. Her criticisms were broad and scathing, but not wholly unjust. The deportment and general bearing of the doctor in the presence of the patient has much to do with both the immediate effect of the treatment instituted and the remote results of future success in building a practice. The influence of his presence should always be beneficial, but can be marred or even made positively injurious by his demeanor. There is much in suggestive therapeutics, certainly more than we know, or possibly care to acknowledge, and the doctor's air can be made of therapeutic value by judicious care. It is probably the want of tact on the part of the physicians that is most criticised, though good judgment and discretion are often wanting in the sick-room.

The first consideration is that the doctor shall be master of the situation. Any vacillation or hesitancy on his part will be felt by the patient or friends and will mar his future usefulness and prospects. This is best secured by perfect ease of manner, and it should be cultivated until it becomes natural with him, really a second nature. A graceful carriage and urbane presence are desirable, but these are not essential. Confidence in one's self in the ability to control the situation, and in one's mental equilibrium, will inspire the confidence of the patient. This will place the patient at ease, and is a point in the doctor's favor. Faith in the doctor will accomplish wonders, and without it the doctor is seriously handicapped.

The first visit of the young doctor to a family is a trying one—more so if it happens to be a prominent family and strangers. He is under close observation and everything he does, from the way he manages his feet and hands to the examination of his patient, is noted and criticised. He should therefore be very careful to neglect nothing that will benefit his patient, and try to avoid giving offense by his manners.

Greetings to the family and a few words of pleasantry to the patient except in very serious cases or of severe pain are admissable before proceeding to an examination of the patient. A few minutes spent in general conversation accustoms the patient to the presence of the doctor. There is often, even with the family physician of several years' standing, an undercurrent of nerve tension with the patient on the arrival of the physician, that should be quieted before proceeding with the examination. Close observation will detect this in the expression of the patient's countenance and it is very frequently noticed in the character of the pulse. It has been our custom for many years to count the pulse of patients twice at least, generally the first and last acts of examination. When this nervous tension is very pronounced it is well to interject into the first part of the examination matters on other questions. Combine general conversation with an occasional question pertaining to the illness until the patient is fully at ease. We have found it often advantageous to suspend the direct examination of a patient, and engage in a few minutes conversation with friends present or even with the patient.

The doctor should be seated near enough to the patient that conversation may be carried on easily, and where the light will be on the patient's face. Do *not* sit on the edge of the bed occupied by the patient. If the patient is at ease, proceed to get the history of the case, if a first visit, or otherwise the conditions and symptoms observed since last visit. The questions should be direct and pointed, and insist upon like answers. This last will be almost imperative in many patients, and much cross-questioning be required. Such are unsatisfactory, but no impatience can be manifested. Often it will be necessary to seek information from other parties, and occasionally out of the hearing or presence of the patient. This is nearly always so in children and young people.

The tone of the voice should be smooth and modulated to suit the character of the patient, the distance between you and the general surroundings. The doctor should keep the patient's interest centered upon the fact that it is his purpose to ascertain the conditions present and the cause of the illness. He should show by his manner and the care with which he proceeds with the examination that he is in earnest in the matter, and that it is for the benefit of the patient. He may be able to make a diagnosis at a glance, or with but little effort, but the young doctor can not afford to stop with a cursory examination. Such a course is detrimental to a well-established practitioner and would be disastrous to the young doctor trying to build up a practice.

Rough jokes may be appreciated by some patients, but the sick-room is not the place for them and the doctor is not the one to either repeat or perpetrate them. The repetition of some anecdote or a bit of humor may be admissable when it is desired to change the current of thought in the patient, but judgment and discretion in their use is very essential. Undue familiarity in the examination of a patient is to be avoided. The patient must be impressed with the importance of medicine as a science, and a disposition of levity and familiarity on the part of the doctor will not do it. Obscene language and vulgar allusions are much worse, and should never be indulged in. These are considered admissable in many sections while in attendance upon obstetrical cases, but the doctor who avoids them will be the more respected.

Sympathy for the patient and especially for great suffering is always appreciated. This, though, may be overdone and is sometimes very much misplaced. It is highly injurious to the very nervous and hysterical patient, and great care should be exercised in giving expression to words of sympathy to these classes. Tenderness in the conversation and in the handling of their patients is their due, and the doctor should not fail to give it to them.

The physical examination of the patient should be thorough, but no unnecessary examinations should be made. Most patients, and especially females, object to exposure, and this should be respected when a diagnosis can be made without it. When necessary, it is better to proceed with it as a matter of course, and there is nothing else to be done. Yet with this the patient and friends will appreciate any efforts on the part of the doctor to make the exposure in as small a degree as possible.

The writing of the prescription or the preparation of the medicines requires great care on the part of the young doctor, for this is the part in which centers the greatest interest of the patient and family. A few moments should be spent in rapidly reviewing the symptoms and making the diagnosis. The directions for administering the remedies and the instructions as to the hygienic care of the patient, diet and nursing, should be clear and explicit. As few words as possible should be used and given as if there was no doubt as to being implicitly followed. In no position can the doctor command more respect than in the manner of delivering his instructions for the care of the patient, and nowhere will hesitancy and vacillating be more disastrous. He has made the examination, arrived at his diagnosis, planned his treatment, and now must show confidence in his judgment. A dictatorial spirit is not best, nor will direct orders be appreciated. Firmness and a spirit of assurance are essential.

These instructions and directions should be given the last thing before leaving. Combining social visits with professional work is rarely advisable. If, for any cause, it is necessary or desirable to prolong the stay of the doctor the instructions for nursing and administering the medicines should be deferred until he is ready to leave. In his leave-taking he should be as positive, decided and as business-like as in any other part of his visit. To bid part of the family good-day, and stop for further conversation, or start out and return for an after-word shows an illy ordered mentality, and ought to be avoided. When ready to leave, leave with a short ceremony—well-wishes for the patient's recovery and announcement of time of next visit are all the ceremonies needed.

With all this it is not well to appear in a hurry—especially in the examination. Thoroughness is of more importance than a few minutes time gained. The point to be made is to leave as soon as the work is finished.

When possible to do so, the family's convenience as to time of visit should be consulted, and an effort be made to meet its wishes. Promptness in meeting such engagements is essential, and apologies or explanations must be offered for failure to do so.

The question of giving the prognosis of the case to the patient must be settled with each individual case. In cases of doubtful recovery, it may not be best to let the patient know of the seriousness of the illness, but some member of the family should be informed of the dangers. This should be done for the benefit of the patient or the family and also for the protection of the doctor.
—*Carolina Medical Journal.*

